# Predicting ICU Mortality in Rheumatic Heart Disease: Comparison of XGBoost and Logistic Regression

**DOI:** 10.3389/fcvm.2022.847206

**Published:** 2022-02-28

**Authors:** Yixian Xu, Didi Han, Tao Huang, Xiaoshen Zhang, Hua Lu, Si Shen, Jun Lyu, Hao Wang

**Affiliations:** ^1^Department of Anesthesiology, The First Affiliated Hospital of Jinan University, Guangzhou, China; ^2^School of Public Health, Xi'an Jiaotong University Health Science Center, Xi'an, China; ^3^Department of Clinical Research, The First Affiliated Hospital of Jinan University, Guangzhou, China; ^4^Department of Cardiovascular Surgery, The First Affiliated Hospital of Jinan University, Guangzhou, China; ^5^Department of Radiology, Medical Imaging Center, The First Affiliated Hospital of Jinan University, Guangzhou, China

**Keywords:** MIMIC-IV, rheumatic heart disease, XGBoost, logistic regression, intensive care unit, mortality, prediction

## Abstract

**Background:**

Rheumatic heart disease (RHD) accounts for a large proportion of Intensive Care Unit (ICU) deaths. Early prediction of RHD can help with timely and appropriate treatment to improve survival outcomes, and the XGBoost machine learning technology can be used to identify predictive factors; however, its use has been limited in the past. We compared the performance of logistic regression and XGBoost in predicting hospital mortality among patients with RHD from the Medical Information Mart for Intensive Care IV (MIMIC-IV) database.

**Methods:**

The patients with RHD in the MIMIC-IV database were divided into two groups retrospectively according to the availability of data and its clinical significance based on whether they survived or died. Backward stepwise regression was used to analyze the independent factors influencing patients with RHD, and to compare the differences between the two groups. The XGBoost algorithm and logistic regression were used to establish two prediction models, and the areas under the receiver operating characteristic curves (AUCs) and decision-curve analysis (DCA) were used to test and compare the models. Finally, DCA and the clinical impact curve (CIC) were used to validate the model.

**Results:**

Data on 1,634 patients with RHD were analyzed, comprising 207 who died during hospitalization and 1,427 survived. According to estimated results for the two models using AUCs [0.838 (95% confidence interval = 0.786–0.891) and 0.815 (95% confidence interval = 0.765–0.865)] and DCA, the logistic regression model performed better. DCA and CIC verified that the logistic regression model had convincing predictive value.

**Conclusions:**

We used logistic regression analysis to establish a more meaningful prediction model for the final outcome of patients with RHD. This model might be clinically useful for patients with RHD and help clinicians to provide detailed treatments and precise management.

## Introduction

Rheumatic heart disease (RHD) is a high priority in areas with restricted health systems (1–3), and causes approximately 250,000 deaths worldwide annually. RHD is heart damage caused by an abnormal immune response to group A streptococcal infections, which makes the morbidity and mortality of its complications a major burden for developing countries (4). Therefore, the early identification and diagnosis of RHD are very important for providing clinicians with meaningful information and allowing timely and appropriate treatment to improve survival outcomes. The pathogenesis of RHD is complicated and not yet fully understood. For this reason, an effective and reliable model for evaluating the prognosis of RHD is urgently needed to offer a basis for comprehensive diagnoses and treatments of the disease and the effective use of medical and health resources. Recent studies have indicated that serum-related markers, such as IL-1β, IL-8, IL-6, CXCL-1, tumor necrosis factor α, antistreptolysin, antideoxyribose, and nuclease B concentrations have been widely used in prognostic evaluations of RHD. However, their prognostic values are limited, for they lack sensitivity or specificity (5, 6). XGBoost is a machine learning technology with the noteworthy characteristics of assembling weak prediction models, and providing flexible and efficient missing data processing for establishing accurate prediction models (5). A logistic regression model is usually developed by the variables of each patient that predict the outcomes of future patients. Its accuracy is primarily based on the ability of the model to correctly assign patients as higher risk. The ability of the model to determine and assign the correct average absolute risk level is essential for judging the usefulness of any new predicting tool (7). Compared with machine learning technology, logistic regression has indicated better predictive performance (8–11).

In a word, this study has two purposes: (I) to use the XGBoost algorithm and logistic regression to compare the overall performance of the model in predicting mortality of patients from the Medical Information Mart for Intensive Care IV (MIMIC-IV) database with RHD during hospitalization, and (II) to perform decision-curve analysis (DCA) and calculate clinical impact curves (CICs) to verify the logistic regression model.

## Methods

### Database

This study was based on version 0.4 of the MIMIC-IV database and contained information on Acute Medical Unit (AMU) and ICU admissions at the Beth Israel Deaconess Medical Center from 2008 to 2019 (12). We extracted patient parameters, including demographic data, vital signs, and laboratory test data. GitHub was used to locate the codes for data extraction (https://github.com/mit-lcp/mimic-iv).

### Study Population

This study included adult patients clinically diagnosed with RHD. The inclusion criteria included (I) patients were elder than 18 years and (II) diagnosed with heart tissue lesions caused by rheumatic fever activity that affected the heart structure. Only patients with first admission information showing RHD would be selected. We used the R packages “vim” (13) and “MICE” (14) for multiple imputation and visualization of <20% missing or randomly missing data in order to improve the accuracy of the review of the MIMIC-IV database, and deleted variables that were absent in more than 20% of observations.

### Data Extraction

The pgAdmin PostgreSQL (version 1.22.1) and Navicat Premium (version 12.0.28) tools were used to extract the raw data of patients diagnosed with RHD during their first hospital admission. R software (version 3.6.3) was used for the data processing (15). The following demographic data were extracted: age, sex, race, weight, length of hospital stay, and hospital death signs at their first admission. The vital signs of ICU patients were collected within 24 h of admission, including heart rate, blood pressure, body temperature, respiratory rate, Oxyhemoglobin saturation (SpO_2_), heart rhythm, glucose, and urine output. Laboratory indicators were extracted, such as routine blood tests, liver function, kidney function, arterial blood gas analysis, Oxyhemoglobin saturation, blood electrolytes, and coagulation function. The following severity scoring systems were extracted: Charlson Comorbidity Index, systemic inflammatory response syndrome score, Simplified Acute Physiology Score-II, sequential organ failure assessment score, Acute Physiology Score-III (APSIII), Logical Evaluation System for Organ Dysfunction, sepsis, chronic kidney disease (CKD), and Glasgow Coma Scale (GCS) score (5). Information on whether the patients were using vasoactive drugs or antibiotics and taking the valve replacement/valvuloplasty, and their ventilation statuses, were all extracted at the first hospital admission.

### Statistical Analysis

Patients with RHD were divided into two groups according to survival or death during hospitalization, and the variables were compared between the groups. We eliminated the confounding variables and outliers with great impacts, and determined the influence of variables on the mortality of patients with RHD during hospitalization using correlation analysis. The Kolmogorov-Smirnov test was used to test continuous variables that conformed to a normal distribution. Student's *t*-test, one-way analysis of variance, Mann-Whitney U test, or Kruskal-Wallis H test were used to test and compare nonnormally distributed continuous data. Categorical variables were reported as numbers or percentages, and were evaluated using chi-square or Fisher's exact tests according to the number of patients.

We developed the logistic regression and XGBoost algorithm models during the model construction phase. First, through backward stepwise analysis and identification using the chi-square test, the variables for which *p* < 0.05 were selected for the logistic regression model. Second, the XGBoost model (16, 17) was established to analyze the impact of each factor on mortality gain during the hospitalization period. Backward stepwise analysis was performed according to the Akaike information criterion (18), variables for which *p* < 0.05 were selected, and clinical symptoms, signs, and laboratory test variables were used to develop the XGBoost machine learning models. We tested and compared the overall performances of the two predictive models using the area under the receiver operating characteristic curve (AUC) and DCA, and selected and verified the model with the highest diagnostic value and prognostic evaluation. Finally, the CIC and DCA were drawn to clarify the clinical practicality and applicability of the model with the highest prognostic value.

Both models were analyzed using R software, and the criterion for statistically significance was set at *p* < 0.05.

## Results

### Baseline Characteristics

**Figure 1:**
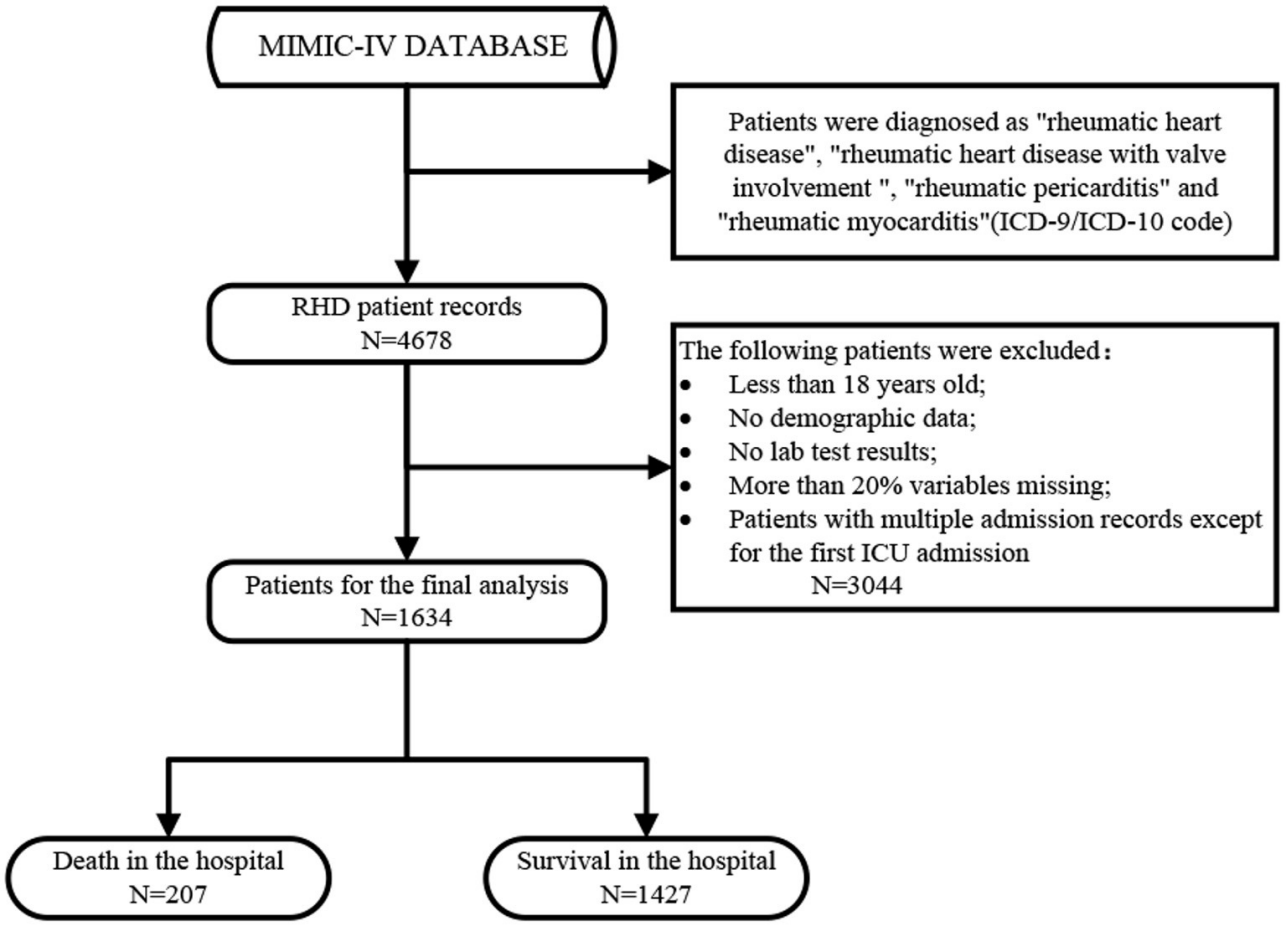
The detailed process of data extraction.

**Figure 2:**
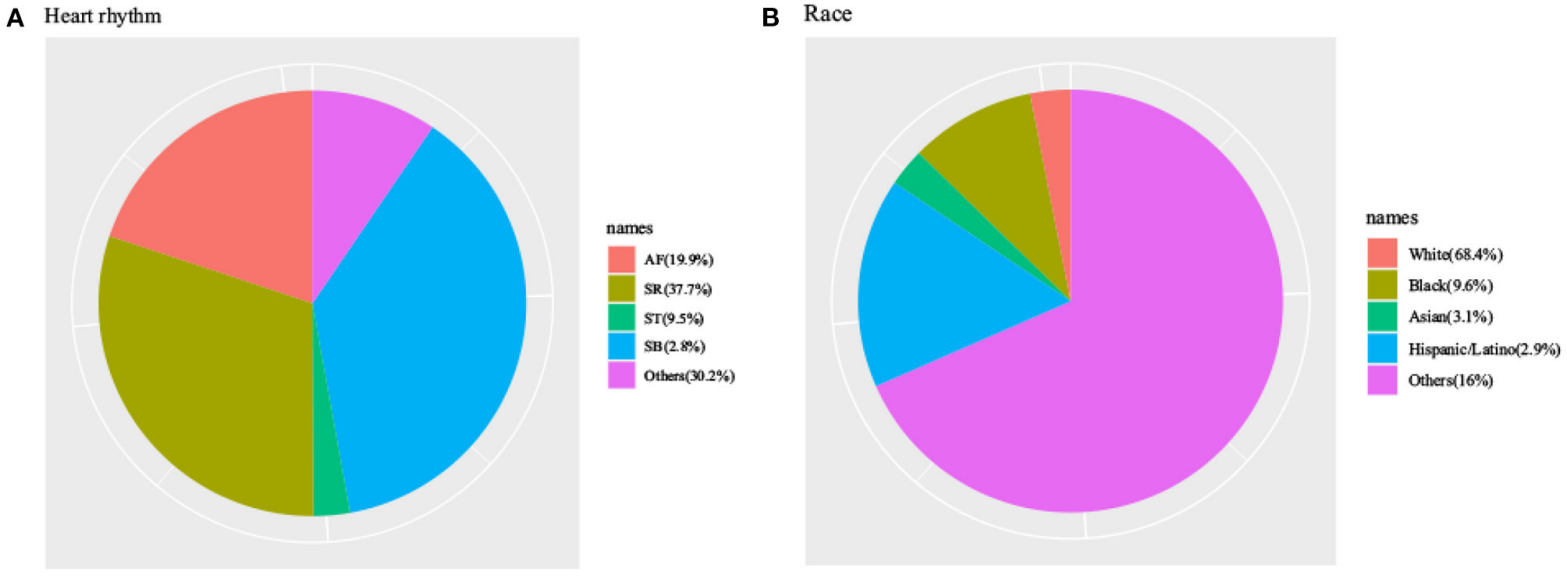
Characteristics of MIMIC-IV patients with Rheumatic heart disease by heart rhythm **(A)** and characteristics of MIMIC-IV patients with Rheumatic heart disease by races **(B)**.

This study analyzed the data of 1,634 patients with RHD, comprising 207 who died during hospitalization and 1,427 survived. Among the dead patients, admission age, norepinephrine, vasopressin, heart rhythm, sepsis, valve replacement, cefazolin, cefepime, mupirocin ointment, vancomycin, CKD, ventilation status, serum creatinine, urine volume, Partial Pressure of Oxygen (PO_2_), total CO_2_, SpO_2_, Partial Pressure of Carbon Dioxide (PCO_2_), blood gas analysis, routine blood test, blood biochemical indicators, scoring system, comorbidity index, and survival group were significant different. However, the differences were not statistically significant (*p* > 0.05) indicators between groups were sex, race, valvuloplasty, PCO_2_, diastolic blood pressure, temperature, weight, bicarbonate, and hematocrit. Figure 1 showed a flow chart of the measures for extracting research objective data. Table 1 compared the baseline characteristics, laboratory data, and vital signs between the dead and surviving patients during the hospitalization periods. Figure 2 was a pie chart showing racial characteristics and overall heart rhythm.

**Table 1:** Baseline characteristics, vital signs, laboratory parameters and statistic results of mimic-IV patients with Rheumatic heart disease.

**Baseline variables and in-hospital factors**		**Survival**	**Death**	***P*-value**
Number (sample size)		1,427	207	
Day		9.0 [6.0, 15.0]	9.0 [5.0, 17.0]	0.422
Sex (%)				
	Man	676 (47.4)	107 (51.7)	0.277
	Female	751 (52.6)	100 (48.3)	
Race (%)				
	White	992 (69.5)	126 (60.9)	0.051
	Black	134 (9.4)	23 (11.1)	
	Asian	42 (2.9)	8 (3.9)	
	Hispanic/Latino	43 (3.0)	4 (1.9)	
	Others	216 (15.1)	46 (22.2)	
Admission_age (year)		74.0 [64.0, 82.0]	78.0 [68.0, 86.5]	<0.001
Weight (kg)		76.6 [64.15, 90.8]	75.5 [63.0, 91.6]	0.396
Vital signs				
Heart_rhythm (%)				
	AF	267 (18.7)	58 (28.0)	0.002
	SR	544 (38.1)	72 (34.8)	
	ST	129 (9.0)	26 (12.6)	
	SB	38 (2.7)	7 (3.4)	
	Others	449 (31.5)	44 (21.3)	
Heart_rate (bpm)		80.4 [72.8, 90.1]	87.4 [75.6, 100.3]	<0.001
Sbp (mmHg)		111.0 [104.0, 119.8]	104.8 [97.9, 114.9]	<0.001
Dbp (mmHg)		59.4 [53.5, 66.0]	58.9 [52.9, 65.6]	0.344
Mbp (mmHg)		74.9 [69.7, 80.8]	72.4 [66.4, 78.8]	<0.001
Resp_rate (bpm)		18.8 [16.8, 21.3]	21.2 [18.4, 24.0]	<0.001
Temperature (°C)		36.7 [36.5, 36.9]	36.7 [36.5, 37.0]	0.61
SpO_2_ (%)		97.1 [95.9, 98.3]	96.6 [95.1, 98.2]	0.003
Ventilation_status (%)				
	No	192 (13.5)	19 (9.2)	0.025
	Oxygen	612 (42.9)	95 (45.9)	
	InvasiveVent	568 (39.8)	76 (36.7)	
	Non-InvasiveVent	36 (2.5)	11 (5.3)	
	HighFlow	19 (1.3)	6 (2.9)	
Urineoutput (L)		150.0 [60.0, 285.0]	105.0 [37.5, 215.0]	<0.001
Laboratory parameters				
Scr		0.8 [0.6, 1.1]	1.1 [0.7, 1.9]	<0.001
PO_2_ (mmHg)		143.0 [58.0, 347.0]	68.0 [41.0, 133.0]	<0.001
PCO_2_ (mmHg)		41.0 [36.0, 46.0]	41.0 [35.0, 49.0]	0.552
PH		7.4 [7.3, 7.5]	7.4 [7.3, 7.5]	<0.001
Baseexcess (mmol/L)		0 [−1.0, 2.0]	−1.0 [−5.0, 1.0]	<0.001
Total co_2_ (mmol/L)		26.0 [24.0, 29.0]	24.0 [21.0, 29.0]	<0.001
Glucose (mg/dl)		128.4 [115.0, 145.5]	138.0 [114.8, 177.3]	<0.001
Rdw		14.6 [13.5, 16.4]	15.9 [14.9, 18.1]	<0.001
White_Blood_Cells (10^9^/L)		9.0 [6.8, 12.6]	10.1 [7.15, 14.9]	0.003
Anion_Gap (mmHg)		26.0 [23.0, 30.0]	29.0 [25.0, 32.0]	<0.001
Bicarbonate (mmol/L)		38.0 [35.0, 41.0]	39.0 [36.0, 42.0]	0.050
Calcium_Total (mmol/L)		10.7 [10.2, 11.3]	10.8 [10.4, 11.6]	0.008
Chloride (mmol/L)		117.0 [115.0, 121.0]	119.0 [116.0, 123.0]	0.001
Hematocrit (%)		51.2 [46.9, 56.1]	52.6 [48.2, 56.8]	0.062
Hemoglobin (g/dl)		17.0 [15.6, 18.8]	17.6 [16.1, 18.9]	0.021
INR_PT		4.4 [3.5, 6.0]	4.8 [3.8, 6.9]	0.013
Magnesium (mmol/L)		3.2 [2.9, 3.7]	3.3 [2.9, 3.8]	0.029
MCH (pg)		37.5 [36.1, 39.0]	38.1 [36.5, 40.0]	0.001
MCHC(g/L)		36.9 [36.3, 37.4]	37.1 [36.4, 37.8]	0.011
MCV (fl)		113.0 [109.0,117.0]	114.0 [109.0,120.0]	0.016
Phosphate (mmol/L)		7.7 [6.5, 9.2]	8.6 [7.4, 10.3]	<0.001
Platelet_Count (10^9^/L)		702.0 [590.5, 856.0]	739.0 [624.0, 884.0]	0.035
Potassium (mmol/L)		6.4 [5.8, 7.4]	6.8 [6.0, 7.7]	0.003
PT (s)		46.2 [36.6, 62.7]	49.8 [38.7, 68.9]	0.030
PTT (s)		150.0 [123.1, 150.0]	150.0 [150.0, 150.1]	0.006
Red_Blood_Cells (10^9^/L)		5.5 [5.2, 5.8]	5.6 [5.2, 5.9]	0.012
Score system				
GCS		14.0 [14.0, 15.0]	12.0 [7.0, 14.0]	<0.001
SOFA		5.0 [3.0, 8.0]	9.0 [5.0, 12.0]	<0.001
Charlson_Comorbidity_Index		6.0 [5.0, 8.0]	8.0 [6.0, 10.0]	<0.001
APSIII		41.0 [32.0, 53.0]	71.0 [55.0, 92.5]	<0.001
LODS		5.0 [3.0, 6.0]	8.0 [6.0, 11.0]	<0.001
MELD		10.0 [10.0, 20.0]	21.32 [10.0, 30.3]	<0.001
SAPSII		37.0 [30.0, 44.0]	48.0 [39.0, 58.0]	<0.001
Advanced life support				
Valve_replacement (%)				
	No	853 (59.8)	183 (88.4)	<0.001
	Yes	574 (40.2)	24 (11.6)	
Valve_shaping (%)				
	No	1,379 (96.6)	204 (98.6)	0.205
	Yes	48 (3.4)	3 (1.4)	
CeFAZolin (%)				
	No	889 (62.3)	183 (88.4)	<0.001
	Yes	538 (37.7)	24 (11.6)	
CefePIME (%)				
	No	1,249 (87.5)	120 (58.0)	<0.001
	Yes	178 (12.5)	87 (42.0)	
Mupirocin_Ointment (%)				
	No	1,158 (81.1)	196 (94.7)	<0.001
	Yes	269 (18.9)	11 (5.3)	
Vancomycin (%)				
	No	816 (57.2)	76 (36.7)	<0.001
	Yes	611 (42.8)	131 (63.3)	
Norepinephrine (%)				
	No	1,059 (74.2)	70 (33.8)	<0.001
	Yes	368 (25.8)	137 (66.2)	
Vasopressin (%)				
	No	1,275 (89.3)	128 (61.8)	<0.001
	Yes	152 (10.7)	79 (38.2)	
Accompanied diseases (comorbidity)				
SIRS		2.0 [2.0, 3.0]	3.0 [2.0, 3.0]	<0.001
Sepsis (%)				
	No	797 (55.9)	65 (31.4)	<0.001
	Yes	630 (44.1)	142 (68.6)	
CKD (%)				
	No	943 (66.1)	98 (47.3)	<0.001
	Yes	484 (33.9)	109 (52.7)	

*AF, Atrial Fibrillation; SR, Sinus Rhythm; ST, Sinus Tachycardia; SB, Sinus Bradycardia; SBP, Systolic Blood Pressure; DBP, Diastolic Blood Pressure; MBP, mean blood pressure; Resp_rate, respiratary rate; SpO_2_, oxyhemoglobin saturation; SCR, serum creatinine; Rdw, Red blood cell distribution width; INR_PT, international normalized ratio; PT, prothrombin time; PTT, partial thromboplastin time; SAPSII, Simplified Acute Physiology Score II; GCS, Glasgow Coma Scale; APSIII, Acute Physiology Score III; LODS, Logistic Organ Dysfunction Score; SOFA, Sequential Organ Failure Assessment; MELD, Model for End-stage Liver Disease; SIRS, Systemic Inflammatory Response Syndrome; CKD, Chronic Kidney Disease*.

### Features Selected in Models

**Figure 3:**
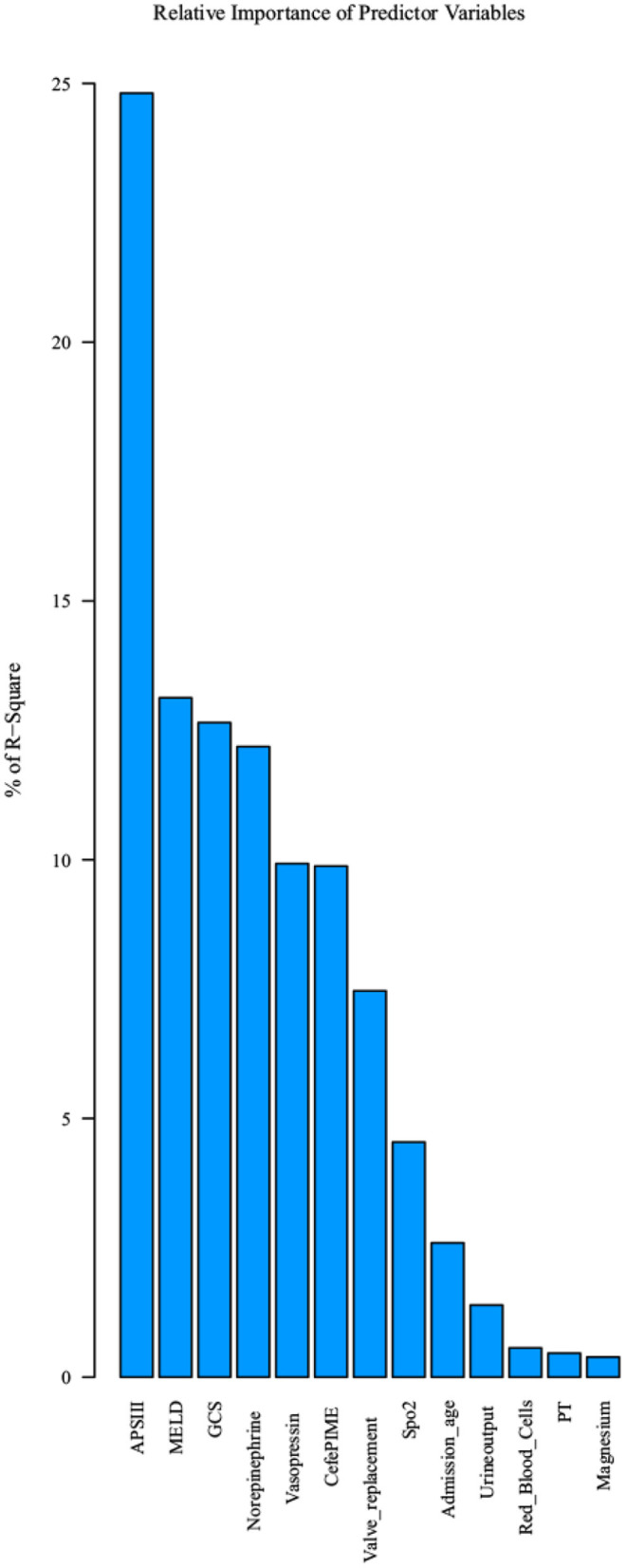
Top 13 features selected using logistic regression and the corresponding variable importance score. X-axis indicates the top 13 weighted variables, Y-axis indicates the importance score which is the relative number of a variable that is used to distribute the data.

The most important features were included in the logistic regression and XGBoost models (Tables 2, 3, respectively), which were determined by the results of backward stepwise regression analysis, and had strong correlations with mortality during hospitalization, with all *p* < 0.05. According to the analysis results for the logistic regression model regarding the contribution rate of each feature (Table 2; Figure 3), the 13 most important variables in the data set were APSIII, vasopressin, Sp_2_, valve replacement, cefepime, GCS score, admission age, Model for End-stage Liver Disease (MELD), urine output, magnesium, prothrombin time (PT), norepinephrine, and red blood cells.

**Table 2:** Features selected in the logistic regression.

**Variables**	**OR**	**CI**	***P*-value**
Norepinephrine	1.091	1.051–1.133	<0.001
Vasopressin	1.104	1.052–1.158	<0.001
Valve_replacement	0.942	0.910–0.975	0.001
CefePIME	1.092	1.048–1.138	<0.001
Mupirocin_Ointment	0.968	0.929–1.008	0.114
CKD	0.970	0.934–1.008	0.116
APSIII	1.003	1.002–1.004	<0.001
Charlson_Comorbidity_Index	1.006	0.999–1.014	0.102
GCS	0.989	0.983–0.996	0.001
SBP	0.999	0.998–1.000	0.109
Resp_rate	1.004	1.000–1.008	0.050
SpO_2_	0.990	0.983–0.997	0.004
Admission_age	1.001	1.000–1.002	0.029
MELD	1.005	1.002–1.007	<0.001
Urineoutput	1.000	1.000–1.000	0.018
Magnesium	0.996	0.993–1.000	0.042
PT	0.999	0.999–1.000	0.016
Red_Blood_Cells	0.964	0.938–0.991	0.010

*OR, Odds Ratio; CI, Confidence Interval; CKD, Chronic Kidney Disease; APSIII, Acute Physiology Score III; GCS, Glasgow Coma Scale; SBP, Systolic Blood Pressure; Resp_rate, respiratary rate; SpO_2_, oxyhemoglobin saturation; MELD, Model for End-Stage Liver Disease; PT, Prothrombin Time*.

**Table 3:** Features selected in the XGboost model.

**Variables**	**OR**	**CI**	***P*-value**
Day	0.997	0.996–0.998	<0.001
Race	1.010	1.001–1.019	0.036
Norepinephrine	1.091	1.051–1.132	<0.001
Vasopressin	1.105	1.054–1.159	<0.001
Valve_replacement	0.948	0.916–0.982	0.003
CefePIME	1.102	1.058–1.149	<0.001
Mupirocin_Ointment	0.966	0.927–1.006	0.093
CKD	0.969	0.933–1.007	0.109
APSIII	1.003	1.001–1.004	<0.001
PCO_2_	0.996	0.991–1.000	0.071
PH	0.476	0.219–1.034	0.061
Baseexcess	1.011	0.999–1.024	0.081
Charlson_Comorbidity_Index	1.009	1.002–1.017	0.017
GCS	0.989	0.982–0.995	0.001
SBP	0.999	0.998–1.000	0.035
Resp_rate	1.005	1.001–1.009	0.027
SpO_2_	0.990	0.983–0.997	0.005
Admission_age	1.001	1.000–1.002	0.129
MELD	1.005	1.002–1.007	<0.001
Urineoutput	1.000	1.000–1.000	0.022
Magnesium	0.997	0.993–1.000	0.072
PT	0.999	0.999–1.000	0.056
Red_Blood_Cells	0.968	0.942–0.996	0.024

*OR, Odds Ratio; CI, Confidence Interval; CKD, Chronic Kidney Disease; APSIII, Acute Physiology Score III; GCS, Glasgow Coma Scale; SBP, Systolic Blood Pressure; Resp_rate, respiratary rate; Spo_2_, oxyhemoglobin saturation; MELD, Model for End-stage Liver Disease; PT, Prothrombin Time*.

### Model Comparisons

**Figure 4:**
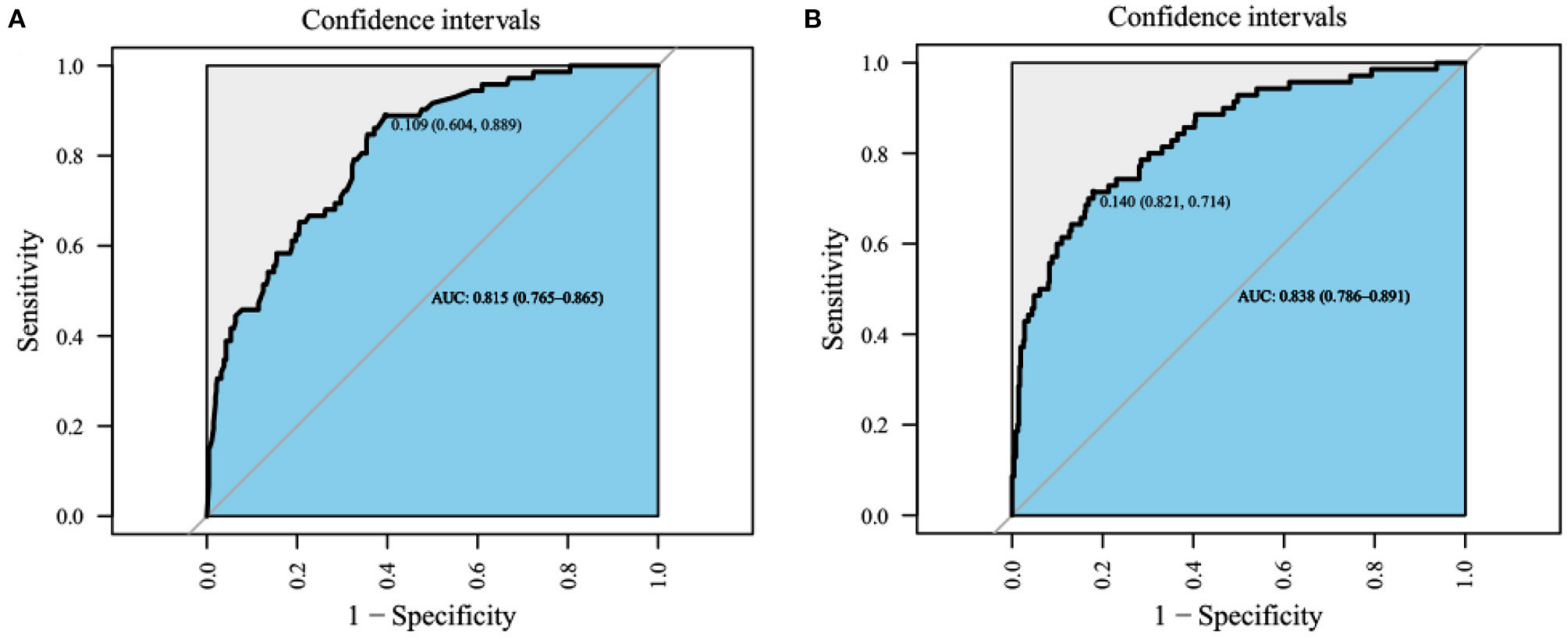
The receiver operating characteristic (ROC) curves. **(A)** XGboost model, AUC is 0.815 [0.765–0.865], **(B)** logistic regression model, area under curves (AUC) is 0.838 [95% confidence interval (CI); 0.786–0.891], the best performance of the models was the logistic regression model.

**Figure 5:**
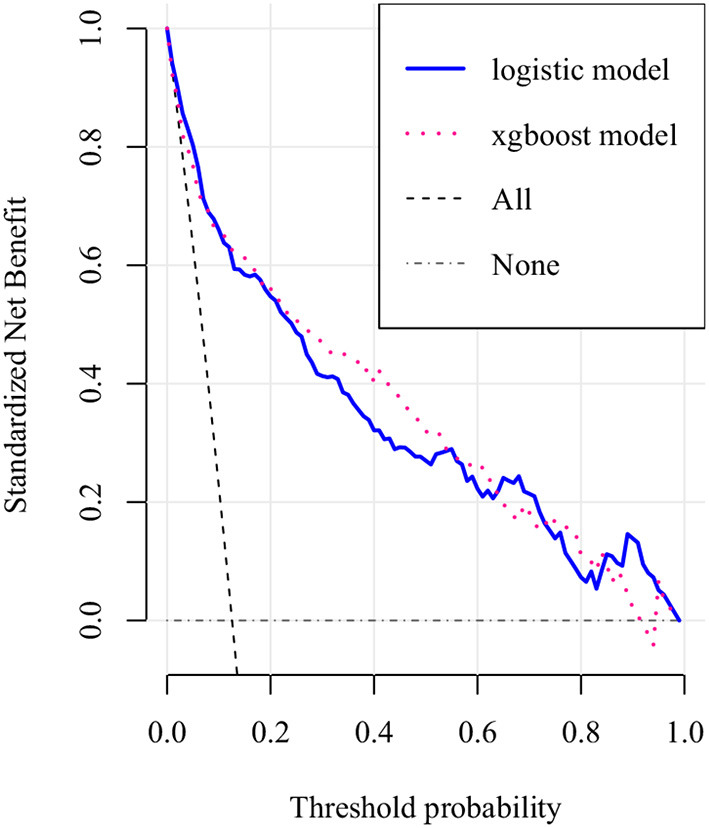
Decision curve analysis (DCA) of the two prediction models. The net benefit curves for the two prognostic models are shown. X-axis indicates the threshold probability for critical care outcome and Y-axis indicates the net benefit. dashed purple line = XGboost model, dashed blue line = logistic model. The preferred model is the logistic model, the net benefit of which was larger over the range of XGboost model.

The AUCs of the two models during the model development and verification stages were 0.838 (95% confidence interval = 0.786–0.891) and 0.815 (95% confidence interval = 0.765–0.865), respectively, which indicated good distinguishing ability (Figure 4). The logistic regression model had a larger AUC than the XGBoost algorithm model. DCA of the two predictive models at the same time indicated that the logistic model had greater net benefit than the XGBoost algorithm model (Figure 5).

### Optimal Model Analysis

**Figure 6:**
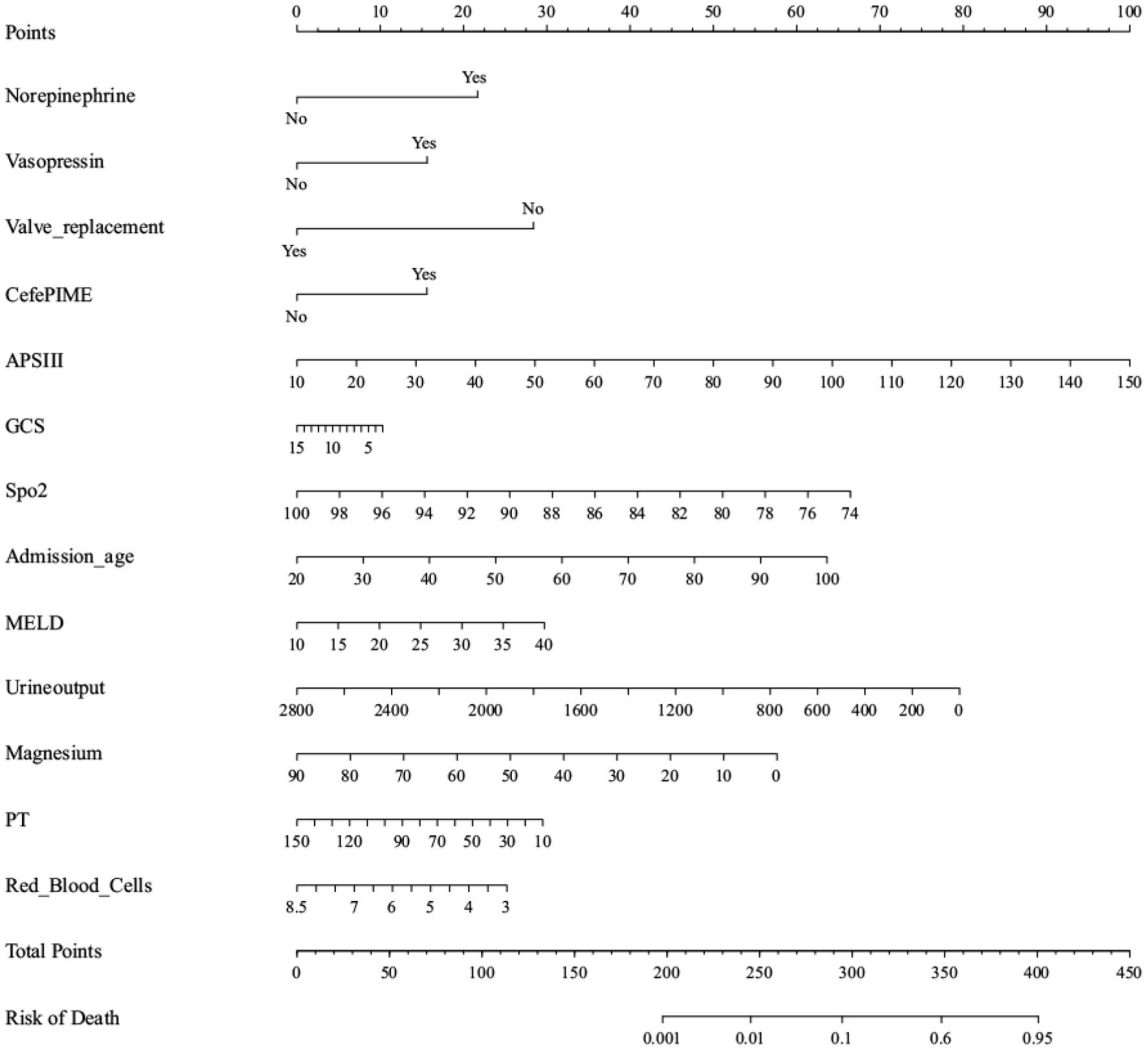
Nomogram to estimate the risk of mortality in Rheumatic heart disease patients. To use the nomogram, we first draw a line from each parameter value to the score axis for the score, the points for all the parameters are then added, finally, a line from the total score axis is drawn to determine the risk of mortality on the lower line of the nomogram.

**Figure 7:**
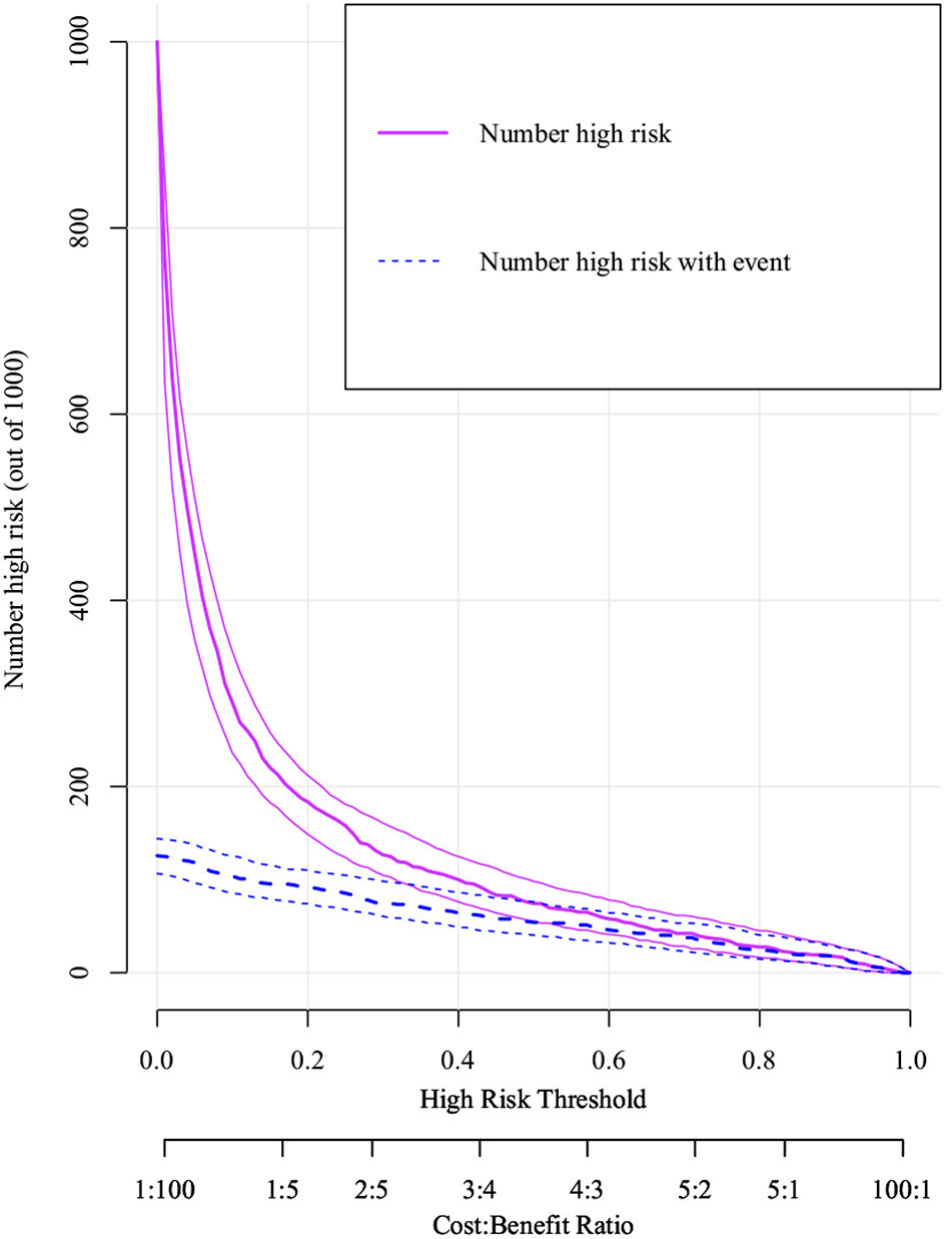
Clinical impact curve (CIC) of logistic model. The purple curve (number of high-risk individuals) indicates the number of people who are classified as positive (high risk) by the model at each threshold probability; the blue curve (number of high-risk individuals with outcome) is the number of true positives at each threshold probability. CIC visually indicated that nomogram conferred high clinical net benefit and confirmed the clinical value of the logistic model.

Figure 6 visualizes the logistic regression model as a nomogram for calculating the risks of mortality and incidence during hospitalization using 13 selected variables for the logistic regression and 15 for the XGBoost algorithm model. The clinical applicability of the risk prediction nomogram in Figure 7 was assessed using CIC analysis. CIC intuitively indicated that the clinical intervention guided by the nomogram scoring system had a superior overall net benefit within the actual range of the threshold probability and affected the prognoses of patients. This indicated that the logistic regression model had significant predictive value. In short, logistic regression analysis was the best model for the prognoses of patients with RHD.

## Discussion

RHD causes severe mortality and huge medical economic burdens in developing countries worldwide (19). Previous reports have indicated that about 1% of over one million asylum seekers who immigrated to Europe in 2015 may have had RHD (20). Previous data suggested that the hospitalization rate of heart disease patients due to RHD increased from 20 to 50% in 1945 and 1963, respectively. According to the annual mortality rate of 1.5%, the number of worldwide deaths from RHD is estimated to be 233,000–294,000 (19).

It is currently difficult for ICU doctors to predict the adverse clinical consequences of patients with RHD, and it is also difficult to enhance the prognoses of these patients through timely interventions and treatments. Hence, the establishment of a reliable clinical prediction model is particularly important in clinical decision-making. The calculated AUCs and DCA suggested the benefits of using the logistic regression model very much more than XGBoost machine algorithm model analysis, which could be used for early predictions of patient mortality during ICU stay.

### XGBoost Model Performance

XGBoost is an efficient, flexible, and scalable machine learning algorithm classifier that improves the subsampling rate, learning rate, and maximum tree depth to control overfitting and enhance its performance. It has been extensively used to detect cardiovascular diseases and associations between prognoses (21). For example, Rong et al. (22) observed that T-wave repolarization synchronization was an important factor for determining the presence of ischemic heart disease using the non-invasive XGBoost machine learning algorithm, and found the correlation between magnetic pole characteristics and cardiac ischemia. Localized ischemia provided an opportunity, and Baskaran et al. (23) used machine learning to obtain insight into the role of images and clinical variables in predicting obstructive coronary artery disease and revascularization, while Tseng et al. (24) determined the risks after cardiac surgery, which could optimize postoperative treatment strategies and minimize postoperative complications.

### Logistic Regression Model Performance

Logistic regression technology has recently been used to analyze the unique benefits of utilizing quantified independent variables, and to determine the influence of a set of independent variables on the regression results (25). Moreover, a prediction model derived from the data set of patient information can predict adverse clinical consequences (26). Sandfort et al. (27) found a significant correlation between long-term heart rate increase and the decrease in ICU survival rate based on logistic regression analysis. Liu et al. (28) investigated the relationship between blood urea nitrogen (BUN) and hospital mortality in patients with critical cardiogenic shock. Their use of a logistic regression algorithm indicated that higher BUN was associated with poorer clinical outcomes. Sun et al. (29) used a logistic regression algorithm to determine that the anion gap was an independent risk variable for the mortality of inpatients in coronary care units (CCUs) and was linked to the poor prognosis of CCU patients. Li et al. (30) used a logistic regression algorithm to estimate whether β2-agonist inhalation would increase the ICU mortality of patients with heart failure. However, compared with other types of predictive models, none of the above studies verified the superiority of the logistic regression model or analyzed it in-depth. More importantly, the main aims of these studies were to detect the poor prognosis of patients with cardiogenic shock and coronary heart disease, whereas logistic regression analysis had not been applied previously to the prognoses of patients with RHD.

### Predictors of Logistic Regression Model Outcomes

The features in the logistic regression and XGBoost models were consistent, highlighting the excellent performance of the logistic regression model. However, the characteristics and adverse consequences of RHD had not yet been completely explained. Therefore, it is necessary to further investigate the effects of these characteristics on patients with RHD. Among these characteristics, ASPIII had the greatest weight, indicating that it was the most important predictor of the RHD mortality of patients in the MIMIC-IV. Meng et al. (31) reported that APSIII and GCS scores were validated disease severity and mortality prediction tools that did well in identifying high-risk patients in a timely manner and in formulating intervention strategies (32). The MELD score was a clinical predictor in the logistic regression analysis of hospital mortality patients with RHD in ICUs and was used to assess cardiovascular-disease-related secondary liver dysfunction (33), and also was taken as a measure of liver and kidney dysfunction. Quantitative indicators had high specificity and sensitivity for predicting the operative mortality of RHD valve surgery (34).

Dzimiri et al. (35) reported that the density of lymphocyte β-adrenergic receptors was markedly decreased in patients with valvular RHD, and the significant decrease in blood oxygen saturation might directly stimulate atrial natriuretic peptide (ANP) release into the heart. There was a good evidence showing that ANP, norepinephrine, vasopressin, and the renin-angiotensin-aldosterone system were involved in controlling water and electrolyte balances. When the blood pressure was low, the elevation of ANP could inhibit the response of vasopressin and renin to arterial hypotension. Hence, the administration of norepinephrine and vasopressin affected the prognosis and mortality of patients in ICUs with RHD, and was essential for the prevention and treatment of complications (36). RHD was the leading cause of valve damage, and heart valve prosthesis implantation was one of the key predictors in the logistic regression model for RHD. Chen et al. (37) and other studies indicated that mitral valve replacement should be performed when patients with RHD had mitral valve damage. Valve replacement could be performed to strengthen driving the decisions making around the surgical treatment of RHD (37). Valve damage that occurring during atrial fibrillation (AF) might be persisting or be aggravated by repeated occurrences, leading to chronic RHD (6). Cefepime is a fourth-generation cephalosporin that is used for the infections of respiratory, urinary, skin, soft tissue and so on from bacteria. Long-term antibiotics use for secondary prevention is critical for preventing disease progression. Prospective studies have demonstrated that life-threatening allergic reactions are uncommon after intravenous antibiotics, and the long-term benefits of this preventive measure outweigh its risks (38). The prevention and treatment of AF recurrence with long-term antibiotics can contribute to reducing the progression and severity of RHD (6, 39).

Age was included in our model as a demographic parameter. Agenson et al. (40) indicated that advanced age had an adverse effect on mortality. In sub-Saharan Africa, RHD is thought to be responsible for up to 32% of heart failure cases (41). Patients with RHD, like those with congestive heart failure, show a significantly improved condition after treatment with cardiac drugs and diuretics, which can be relieved by treating pulmonary vein congestion that can prolong diastolic time and improve cardiac output (6). For this reason, recording urine volume is of great significance to ICU patients (42).

PT was another key predictor in the logistic regression model. Increased fibrinogen levels in patients with RHD were linked to accidental ischemic stroke (43). Arvind and Ramakrishnan (6) suggested that patients with RHD accompanied by AF, a medical history of thromboembolism, or left atrial thrombosis require anticoagulant therapy when testing their coagulation function indexes, including PT. The most common persistent arrhythmia is AF, which can increase the likelihood of stroke about 5 fold and the risk of death from all causes by 2 fold in patients with RHD.

Serum magnesium is a particularly interesting parameter. With serum magnesium levels >3.8 mg/dl, sinus rhythm of patients had a conversion rate of 88.89%, which far exceeded that of 16.67% in patients with serum magnesium levels <3.8 mg/dl. Intravenous magnesium supplementation can significantly improve the effect of converting AF and improve sinus rhythm in patients with RHD (44). A recent study by Deora et al. (45) found that the main cause of heart failure was diastolic heart failure, and the most common cause was RHD. Anemia has a huge impact on the course and prognosis of patients with heart failure. Appropriate interventions and prognoses should be provided early in the course of the disease. Treatment will improve the survival rate of patients, and the red blood cell count has also been included in the model (45). Our research indicated that in addition to the unknown heart rhythm and normal heart rhythm, AF accounted for the highest proportion of cases. To prevent adverse complications such as cardiovascular or cerebrovascular embolism, some studies have indicated that socioeconomic and environmental factors related to race were also the reasons for the increase in RHD prevalence (46).

### Strengths

The main advantage of this study was that it was the first one to use a logistic regression model to predict hospital mortality of patients with RHD from the MIMIC-IV database. According to the variables selected by the backward stepwise regression analysis, the accuracy and representativeness were improved over XGBoost. The models were compared and verified using DCA and CIC, and certain important parameters such as the body weight and heart rhythm were not missing.

### Limitations

Our research inevitably has some limitations: Firstly, it was a retrospective observational study, not a randomized study, and the data were only obtained from the MIMIC-IV database, which the majority of patients were white, and there may be unobserved confounding factors that might lead to potential biases in the results. Secondly, the MIMIC-IV database did not provide patient history and long-term follow-up events. The inherent limitations in the data extraction technology meant that some crucial influencing variables were overlooked. Thirdly, RHD patients might also have complications such as cardiovascular and cerebrovascular accidents during ICU admission. Fourthly, despite the very high quality of the MIMIC-IV database, there were still some underlying variables and missing data that prevented the availability of certain clinical variables and surgical operation data, including left ventricular ejection fraction, functional classification of heart failure, echocardiographic data, variables such as C-reactive protein, lactate and D-dimer, implying that the inability to accurately identified the severity of RHD is another limitation of this study. Finally, as a retrospective observational single-center study of electronic health record data, the earliest cases were taken from nearly 14 years ago, when care might have been inconsistent with currently accepted standards. The single-center nature of the study might also make our findings limiting the applicability of other sites. Nonetheless, single-center studies have increased the likelihood that patients will be treated uniformly, alleviating concerns that observed differences in mortality may be due to differences in practice across centers.

Despite owning these limitations, it can currently provide comprehensive and high-quality data on critical illnesses, and has provided a large number of scientific researchers with good research ideas and data, and published many high-quality scientific articles. And some studies using retrospective studies are sufficient, such as when rofecoxib was withdrawn from the market or we convinced the public that smoking was associated with a risk of cancer. In this paper, we aimed to utilize secondary analysis of electronic health record (EHR) data to evaluate practical application-related tests and treatments based on limited benefit or theory, and we considered that the proposed model could help further the understanding of the prognosis of patients with RHD during ICU hospitalization. However, further prospective multicenter studies are needed to validate our findings.

## Conclusions

In summary, this research has indicated that machine learning based on logistic regression analysis algorithm is better than using the XGBoost algorithm. Our simple and efficient nomogram based on logistic regression analysis is indicated to be clinically useful. It can help clinicians to tailor precise management and treatments for patients with RHD, which is conducive to maximizing the survival probability of these patients.

## Data Availability Statement

The raw data supporting the conclusions of this article will be made available by the authors, without undue reservation.

## Author Contributions

YX, DH, and TH performed statistical analysis and data interpretation. HW and JL contributed to the study concept and study design and contributed equally. XZ performed literature research and data extraction. HL and SS were responsible for the quality control of data and algorithms. All authors contributed to writing of the manuscript and approved the final version.

## Funding

This study was funded by National Natural Science Foundation, China (Grant No. 82071340); Natural Science Foundation of Guangdong Province, China (Grant No. 2018A0303131003); Science and Technology Planning Project of Guangdong Province, China (Grant No. 2020A1414010291) and Medical Science and Technology Foundation of Guangdong Province, China (Grant No. A2019550).

## Conflict of Interest

The authors declare that the research was conducted in the absence of any commercial or financial relationships that could be construed as a potential conflict of interest.

## Publisher's Note

All claims expressed in this article are solely those of the authors and do not necessarily represent those of their affiliated organizations, or those of the publisher, the editors and the reviewers. Any product that may be evaluated in this article, or claim that may be made by its manufacturer, is not guaranteed or endorsed by the publisher.

## Abbreviations

ICU: Intensive care unit
RHD: Rheumatic heart disease
MIMIC-IV: Medical Information Mart for Intensive Care IV
AUCs: Areas-under-the-receiver operating characteristic curves
DCA: decision-curve analysis
CIC: Clinical impact curve
AMU: Acute Medical Unit
SpO_2_: Oxyhemoglobin saturation
APSIII: Acute Physiology Score-III
CKD: chronic kidney disease
GCS: Glasgow Coma Scale
PO_2_: partial pressure of oxygen
PCO_2_: Partial Pressure of Carbon Dioxide
MELD: Model for End-stage Liver Disease
PT: prothrombin time
BUN: blood urea nitrogen
ANP: atrial natriuretic peptide
AF: atrial fibrillation
CCUs: coronary care units
ROC: The receiver operating characteristic curves
OR: Odds ratio
CI: Confidence interval.
